# From connection to conservation: Nature‐based mindfulness as a pathway to pro‐environmental behaviors

**DOI:** 10.1111/aphw.70220

**Published:** 2026-09-09

**Authors:** Patricia Zieris, Markus M. Müller, Meike Krebs‐Fehrmann, Katharina Thümer, Peter Loreth, Doris Pokorny, Elisabeth Kals

**Affiliations:** ^1^ Social and Organizational Psychology Catholic University Eichstätt‐Ingolstadt Eichstätt Germany; ^2^ Department of Psychosomatic Medicine and Psychotherapy Paracelsus Medical University, General Hospital Nürnberg Nürnberg Germany; ^3^ UNESCO Biosphere Region Berchtesgadener Land District Government of Upper Bavaria Freilassing Germany; ^4^ UNESCO Biosphere Reserve Rhoen District Government of Lower Franconia, Bavarian Administration Unit Oberelsbach Germany

**Keywords:** emotional affinity toward nature, environmental stewardship, longitudinal study, nature connectedness, nature‐based intervention, nonrandomized controlled trial

## Abstract

Nature‐based mindfulness interventions (NBMIs) are increasingly recognized for fostering nature connection and promoting pro‐environmental behavior (PEB). However, robust longitudinal evidence of their sustained impact within the general population remains limited. This study investigated how such an intervention impacts pro‐environmental attitudes and behaviors. Adults (M_age_ ≈ 46 years, predominantly female) from a German biosphere region were nonrandomly assigned to an experimental group (*n* = 56) undergoing a 3‐week NBMI or to a wait‐list control group (*n* = 49). Participants completed measures of nature connection (nature‐based mindfulness and emotional affinity toward nature), environmental cognition (threat awareness and responsibility), and behavior (intentions, PEB, and time in nature) at three time points over 15 weeks. Mixed models for repeated measures (MMRM) analyses revealed that the NBMI group showed significantly greater increases in nature connection and responsibility attributions post‐intervention than the control group. Although immediate PEB effects were mixed, a key finding was a delayed impact: The NBMI group exhibited significantly greater PEB (*d* = 0.53) and spent more time in nature (*d* = 0.34) at follow‐up compared to T1, consistent with a time‐lagged effect potentially mediated by enhanced connection. These findings suggest that NBMIs effectively cultivate a deeper relationship with nature, providing a psychological foundation for sustained environmental stewardship. Implications and future research directions are discussed.

## INTRODUCTION

The escalating environmental crisis, marked by pollution, extreme weather events, and climate change, poses significant threats to both human well‐being and planetary stability (Eurostat, 2025; Intergovernmental Panel on Climate Change, 2023). Because this crisis is fundamentally rooted in human activities (Clayton et al., 2016; Wuebbles et al., 2017), addressing it necessitates urgent action to cultivate pro‐environmental behaviors (PEBs)—actions that minimize environmental harm and actively contribute to nature preservation (Steg & Vlek, 2009). PEBs align with global initiatives such as the United Nations (UN) Sustainable Development Goal (SDG) 13: Climate Action (Lee et al., 2016), which underscores the need for collective efforts toward ecological sustainability.

A significant barrier to cultivating PEBs lies in humanity's growing disconnection from nature (Kals et al., 2025). Modern lifestyles often separate individuals from natural environments physically and psychologically (Capaldi et al., 2015; Soga & Gaston, 2016), weakening the emotional bonds crucial for conservation efforts (Martin et al., 2020; Pritchard et al., 2020; Shrestha et al., 2025). Fostering effective environmental stewardship—the responsible use and protection of the natural world through conservation and sustainable practices (e.g. Bennett et al., 2018)—therefore requires strategies that address key psychological precursors to action. Beyond fostering an emotional connection, this includes enhancing individuals' cognitive acknowledgment of the issue, such as their awareness of environmental threats, and strengthening their sense of agency and obligation, reflected in their attributions of responsibility for nature conservation. These cognitive and affective factors are established determinants of pro‐environmental engagement (Bamberg & Möser, 2007). To this end, nature‐based interventions (NBIs)—intentional programs engaging people in nature‐based experiences to enhance well‐being and other positive outcomes—have gained considerable attention.

Although NBIs encompass a wide range of activities including nature education, mindful walking, and forest bathing, the integration of mindfulness practices represents a particularly synergistic development, leading to what are termed nature‐based mindfulness interventions (NBMIs). By systematically guiding attention to sensory experiences within the natural world, these interventions aim to cultivate a deeper sense of interconnectedness with the environment. This process can significantly strengthen the affective and cognitive bonds critical for fostering the attitudes and behaviors needed to address ecological challenges (Bamberg & Möser, 2007; Kollmuss & Agyeman, 2002).

This article presents findings from an experimental study investigating how participating in an NBMI fosters pro‐environmental attitudes and increases conservation behaviors. By exploring how guided contact with nature intersects with mindfulness practices to restore human–nature connections and inspire sustainable actions, this research offers valuable insights into strategies for mitigating ecological harm.

### The role of connection in fostering PEBs

A pivotal factor in fostering PEBs is the depth of an individual's connection to the natural world, which extends beyond mere physical contact. Whereas spending time in natural environments provides psychological benefits like stress reduction (Barragan‐Jason et al., 2023; Corraliza et al., 2012; Hossain et al., 2020), inspiring long‐term environmental stewardship requires a deeper emotional connection (Martin et al., 2020). “Connection to nature” is a multifaceted psychological construct (e.g. Tam, 2013) that serves as a key driver of ecological engagement, a link that is well established by meta‐analytic evidence (Mackay & Schmitt, 2019; Whitburn et al., 2020). This connection, which encompasses a spectrum from simple physical contact to deep emotional bonds, is understood through several related theories. Concepts like nature relatedness (Nisbet et al., 2009), nature connectedness (Mayer & Frantz, 2004), emotional affinity toward nature (Kals et al., 1999), and the inclusion of nature in self (Schultz, 2001) all converge on the idea that fostering a meaningful human–nature relationship is crucial for driving PEBs (Tam, 2013). Similarly, the nature‐based biopsychosocial resilience theory proposes that nature experiences build personal resources that deepen these critical bonds (White et al., 2023).

A robust body of research confirms this link. Emotional connection to nature—often cultivated through positive experiences in childhood or adulthood (Kahn & Kellert, 2002; Müller et al., 2009)—is a strong predictor of conservation‐oriented actions (Frantz & Mayer, 2014; Ives et al., 2018; Mackay & Schmitt, 2019; Whitburn et al., 2020). This connection often serves as a key mediator between nature contact and environmentally responsible behaviors (Cleary et al., 2020; Mayer et al., 2009; Richardson, 2023). The causal link between nature connectedness and PEBs is further substantiated by meta‐analytic evidence across diverse populations (Barragan‐Jason et al., 2022; Mackay & Schmitt, 2019).

### Mindfulness in nature as a pathway to connection

Building on initial contact with nature, mindfulness practices offer a powerful means to deepen human–nature relationships. Mindfulness is characterized by paying attention intentionally to the present moment nonjudgmentally (Kabat‐Zinn, 1994). This practice serves as a natural complement to nature engagement by guiding attention to natural stimuli and sensory impressions. It enhances this relationship through three interrelated mechanisms, thereby providing an essential complement to the psychological benefits of physical contact alone (van Gordon et al., 2018). First, mindfulness sharpens sensory awareness by directing attention to present‐moment stimuli such as natural sights and sounds (Bishop et al., 2004; Brown & Ryan, 2003). Second, it facilitates decentering, reducing ego‐driven perspectives and fostering a sense of interconnectedness with the environment (Howell et al., 2011; Schutte & Malouff, 2018). Finally, it promotes openness and acceptance toward natural surroundings, enabling richer emotional bonds by embracing their complexity (Djernis et al., 2019).

Researchers often discuss the integration of mindfulness with nature exposure in the context of attention restoration theory (Kaplan & Kaplan, 1989), which posits that natural environments restore attentional capacity through soft fascination (Capizzi & Kempton, 2023; Pham & Sanocki, 2024). Although restoration is important, the mechanisms of mindfulness in nature extend beyond it, simultaneously strengthening nature connectedness, fostering personal well‐being, and encouraging actions that protect and preserve ecosystems (Unsworth et al., 2016). By enhancing this connection through mindful practices in natural settings, individuals may be more motivated to adopt sustainable behaviors (Barbaro & Pickett, 2016).

### NBMIs

Research on NBMIs has surged in recent years, with studies focusing on their capacity to integrate mindfulness practices with direct immersion in natural environments (Djernis et al., 2023; Vitagliano et al., 2023). Although prior work has established the psychological and behavioral benefits of both mindfulness and nature contact (Capaldi et al., 2015; Capizzi & Kempton, 2023), NBMIs are increasingly recognized for their potential to synergistically strengthen individuals' connection to nature and promote PEBs beyond the effects of either practice alone (Macaulay et al., 2024; Ray et al., 2021). A growing body of experimental and quasi‐experimental studies demonstrates that NBMIs reliably enhance participants' sense of connection to nature. For example, interventions combining guided mindfulness exercises with exposure to natural settings—such as mindful walking, forest bathing, or meditation with nature sounds—have fostered enhanced nature connectedness (Nisbet et al., 2019). This outcome is supported by a meta‐analysis from Schutte and Malouff (2018), which found a significant, moderate positive correlation (*r* = .25) between trait mindfulness and connectedness to nature across 12 samples. These findings extend prior cross‐sectional research and suggest that NBMIs can foster a deeper and more enduring bond with the natural world. Moreover, individuals who participate in NBMIs often report greater motivation to seek out nature experiences in daily life, pointing to a positive feedback loop between intervention participation and ongoing nature contact (Capizzi & Kempton, 2023).

Some critics argue that certain mindfulness applications risk fostering passive acceptance of environmental degradation or individualizing responsibility for systemic ecological issues (Sauerborn, 2022). However, empirical evidence suggests otherwise, highlighting mindfulness as a potential catalyst for active engagement in conservation efforts (Ericson et al., 2014). Evidence linking NBMIs to PEB is accumulating, though findings are nuanced. For instance, Ray et al. (2021) found that a 4‐week online mindfulness meditation program with nature‐based components led to increases in both nature connectedness and PEB. Similarly, a daily diary study reported that mindfulness‐based practices in nature predict both same‐day and subsequent‐day pro‐environmental actions, suggesting a time‐lagged effect (Richter & Hunecke, 2022). Whereas mediation analyses often point to enhanced connection to nature as a key pathway (Hanley et al., 2020; Pereira & Forster, 2015), other research highlights additional psychological mediators. For example, Loy et al. (2022) found that the relationship between mind–body practice and pro‐environmental engagement was explained by practitioners' higher levels of self‐compassion and global identity.

In contrast to studies reporting beneficial effects of NBMIs on PEB, Geiger et al. (2020) found no significant impact of a sustainability‐adapted mindfulness‐based intervention on participants' PEB or their attitudes toward such behaviors. Although the intervention did not close the gap between attitudes and actions, it was associated with a modest reduction in materialistic values, indicating that mindfulness training may influence underlying value orientations that could, over time, support more sustainable consumption patterns. Similarly, Le Houcq Corbi et al. (2024) tested the effects of a 31‐day online mindfulness intervention on actual pro‐environmental choices using incentivized decision tasks with real‐world environmental consequences. Contrary to their hypotheses, the intervention reduced rather than increased participants' preferences for pro‐environmental and prosocial outcomes. No effects were observed on future‐oriented decision‐making or on self‐reported pro‐environmental attitudes. These findings challenge the assumption of a straightforward positive effect of mindfulness training on PEB and suggest that the relationship may be more complex, potentially depending on content and context.

Despite growing evidence supporting NBMIs' benefits, the field is constrained by notable limitations that restrict the robustness of current findings and generalizability. Recent comprehensive reviews call for more research into the antecedents and underlying psychological processes that form nature connection (Lengieza & Swim, 2021) and highlight the need for interventions that can produce sustained, long‐term change (Sheffield et al., 2022).

Within this context, two specific limitations are apparent. First, most empirical studies investigating the relationship among mindfulness, nature exposure, and PEB have predominantly targeted specific subpopulations, such as employees (Kumar et al., 2022; Patel & Holm, 2018) or students (Carlson et al., 2025; Jalón et al., 2022). This narrow focus raises concerns regarding the external validity of these results, as findings from relatively homogeneous groups may not readily extrapolate to the broader population. Moreover, a critical limitation is the predominant emphasis of the extant literature on short‐term outcomes. As Capizzi and Kempton (2023) and Djernis et al. (2023) have observed, most studies assess NBMIs' immediate or near‐term effects, precluding substantive conclusions about their long‐term efficacy or sustainability. This temporal constraint is particularly salient given that maintaining pro‐environmental attitudes and behaviors likely depends on enduring psychological and behavioral change, which only longitudinal research designs with follow‐up assessments can capture (Kwasnicka et al., 2016; Rau et al., 2022).

### The present study

Building on the literature reviewed above, and in response to the identified limitations—particularly the lack of longitudinal studies and the focus on specific populations—the present study adopts a rigorous, longitudinal approach to examine the effects of an NBMI on environmental attitudes and PEB within a general population sample over a 15‐week period. This study was conducted as part of a larger research project evaluating the effectiveness of mindfulness‐based and relaxation‐based nature interventions on mood (primary outcomes) and environmental variables (secondary outcomes) in clinical and nonclinical samples.^1^ The clinical arm of the project, which examined the effects of the interventions on mood in depressed patients, has been published separately (Müller et al., 2025). Here, we focus on the NBMI's impact on environmental attitudes and PEB within the general population from the UNESCO Biosphere Region Berchtesgadener Land, Germany. This study aims to clarify how NBMIs can foster environmental attitudes and PEB, even when these are not the intervention's primary focus.

The primary hypothesis posited that participants in the experimental group would demonstrate significantly greater improvements in environmental attitudes—namely, nature‐based mindfulness, emotional affinity toward nature, awareness of environmental threats, and attributions of responsibility for nature conservation (H_1a_)—as well as pro‐environmental intentions and PEB (H_1b_) compared to the control group between T1 and T2. A secondary objective was to assess the stability of these effects over time. Consequently, the second hypothesis (H_2a_ and H_2b_) examined whether the enhancements observed in the experimental group would persist at the 12‐week follow‐up (T3).

## METHODS

### Study design

This study employed a two‐arm nonrandomized controlled trial. This approach stemmed from ethical considerations, practical constraints due to the COVID‐19 pandemic, and the study's objective. Ethically, this ensured all participants could eventually benefit from the intervention. Practically, the pandemic imposed significant challenges on randomization procedures and participant recruitment. Furthermore, the study aimed to assess the feasibility of NBMIs in real‐world settings. This method was therefore adopted not only for this reason but also to bolster external validity by evaluating the intervention as it might operate within a naturalistic community context where strict randomization can be challenging or less reflective of typical implementation (see Rothwell, 2005, for further discussion on external validity).^2^

### Intervention

The NBMI was designed by UNESCO Biosphere staff in cooperation with specialists in psychosomatic medicine, psychotherapy, and behavioral therapy. The intervention integrated formal and informal exercises from established mindfulness programs like Mindfulness‐Based Stress Reduction and Mindfulness‐Based Cognitive Therapy, including practices such as breathing meditation, the body scan, mindful yoga, and the incorporation of mindfulness into everyday actions. Complementing these mindfulness components, methods of nature education were applied, featuring exercises focused on sharpening sensory awareness in nature, adopting different perspectives, actively experiencing natural surroundings, and mindfully encountering plants and animals.^3^

#### Experimental group intervention

Participants in the experimental group engaged in the NBMI once a week for a 4‐h session over a period of three consecutive weeks. These sessions were conducted from July to November 2021 and in May 2022. To leverage diverse natural environments, sessions took place in five strategically chosen locations within the UNESCO Biosphere Region Berchtesgadener Land, Germany. Group sizes ranged from 4 to 10 participants to facilitate effective intervention delivery while adhering to COVID‐19 safety protocols in place at the time. Each session was led by a member of the biosphere region staff involved in the intervention's design, accompanied by a trained health professional (psychologist or psychotherapist) and a trained forest and nature educator or nature coach. This multidisciplinary leadership team ensured participants received expert guidance on both the psychological and environmental aspects of the NBMI.

#### Control group condition

The control group was designed as a wait‐list control group. During the 3‐week period when the experimental group received the intervention, control group participants did not receive any intervention. Following the completion of the T2 data collection (after the 3‐week period), control group participants were offered a single 4‐h NBMI session as compensation for their participation in the study.

#### Data collection

Data for both groups were collected using paper‐and‐pencil questionnaires administered immediately before the initial session (T1) and immediately after the 3‐week intervention period for the experimental group (or an equivalent 3‐week period for the control group) (T2). For the experimental group, additional follow‐up data were collected 3 months after the final session (T3).

### Participants

We conducted the recruitment and informed consent process systematically to ensure ethical standards and that participants were well informed. Staff of the biosphere region recruited participants through various channels, including adult education centers, press releases, social media platforms, and the project's official website. Interested individuals contacted the project staff at the biosphere region for more information. Assignment to the experimental (intervention) and wait‐list control groups was managed by the biosphere region staff. As intervention dates were fixed and group sizes were limited, applicants were allocated on a first‐come, first‐served basis. Those who applied after the initial intervention slots were filled were placed in the wait‐list control group.

The informed consent process ensured participants were thoroughly briefed on inclusion and exclusion criteria, study procedures, risks, and benefits. Eligible individuals received a consent form to confirm understanding and voluntary agreement to participate and were provided with adequate time for decision‐making.

Inclusion criteria required that participants be at least 18 years old, possess adequate tetanus vaccination,^4^ and own sturdy footwear and weatherproof clothing. Moreover, participants were required to have a good command of German to ensure effective communication and comprehension during sessions. Once recruitment was underway and in response to significant interest from potential participants older than 59, we subsequently removed this initial upper age limit. This decision was based on a careful assessment by relevant experts, including the responsible biosphere region staff, the nature ranger, and the accompanying psychologist or psychotherapist. These professionals evaluated older participants' potential risks and benefits, ensuring the change was made with due consideration for participant safety and study integrity. Removing the upper age limit enhanced inclusivity, allowing a broader range of individuals to benefit from the NBMI.

We established exclusion criteria to ensure the study's safety and validity. These criteria included a history of manic episodes, delusional disorders, or other severe psychiatric conditions. Additionally, we excluded individuals with physical impairments that could limit their mobility in outdoor settings, as well as those unable to provide informed consent.

### Outcome measures

#### Pretest

To verify the quality of the instruments prior to their use in the main study, we conducted a preliminary psychometric validation study. For this purpose, we recruited an ad hoc sample (*N* = 205) from the general population who completed an online questionnaire between February and April 2020. The scales selected for this pretest were based on German‐language versions utilized and adapted in pilot studies that drew upon the following established instruments: *nature‐based mindfulness* (five items, adapted from Michalak et al., 2016), *emotional affinity toward nature* (six items, adapted from Kals et al., 1999), *awareness of environmental threats* (four items, adapted from Montada et al., 2007), *attributions of responsibility for nature conservation* (four items, adapted from Burger, 2013), *pro‐environmental intentions* (five items, adapted from Montada et al., 2007), and *PEB* (three items, own compilation). All scales used a 6‐point Likert scale, ranging from 1 (*does not apply at all*) to 6 (*fully applies*).

Our pretest involved a rigorous analytical approach, including factor analysis, reliability analysis, correlation analysis, multiple regression analysis, and mean comparisons utilizing *t*‐tests (Bruckbauer, 2019). We used SPSS Statistics 29 (IBM Corp., Armonk, NY, USA) to perform these analyses. Based on these psychometric evaluations, we excluded one item related to awareness of environmental threats (“I am concerned when I think about the environment in which our children and grandchildren are likely to live”) from the analysis. All other items demonstrated acceptable psychometric properties (.74 < *α* < .94) and were thus selected for the final instrument used in the current study.

#### Variables

Participants completed the main study outcome measures using self‐report scales and single items. Based on their conceptual focus, we categorized these measures into three groups: nature connection, environmental cognition, and behavior.

##### Nature connection measures

Nature‐based mindfulness was measured using five items (e.g. “In nature, I pay attention to sensations, such as sunshine on my face or wind in my hair”; *α* = .91). Emotional affinity toward nature was measured using six items (e.g. “When I am in nature, I feel safe and secure”; *α* = .96).

##### Environmental cognition measures

Awareness of environmental threats was measured using three items (e.g. “If nothing fundamentally changes, the endangerment of nature will worsen in the next few years”; *α* = .86). Internal attribution of responsibility for nature conservation (e.g. “I feel responsible for actively contributing to nature conservation myself”; *r* = .69) and external attribution of responsibility for nature conservation (e.g. “The government is responsible for doing something for nature conservation”; *r* = .74) were measured using two items each.

##### Behavioral measures

Pro‐environmental intentions were measured using five items (e.g. “I am willing in principle to take measures to protect nature”; *α* = .80). PEB was measured using three items (e.g. “I act in a nature‐friendly way in many respects”; *α* = .94). Current contact with nature was measured using a single item (“I currently spend a lot of time in nature”).

In addition, one item was used to measure contact with nature in childhood (“I spent most of my childhood and adolescence in an urban area”).^5^

#### Sociodemographic data

Sociodemographic data included gender, age (in years), parental status, and prior experience with nature or mindfulness training. Respondents indicated this experience as “yes” or “no” and, if applicable, provided a free‐response answer specifying the training context.

Finally, participants created a self‐generated code to match answers across the three time points. This code used the first letter of the mother's first name, the third letter of the participant's first name, their day of birth, and the first two letters of their city of birth.

### Statistical analysis

We performed statistical analyses using SPSS Statistics 29 (IBM Corp., Armonk, NY, USA) and R (R Core Team, 2020). We used mixed models for repeated measures (MMRM) to examine the trajectories of the dependent variables as a function of time (T1 and T2), group (experimental vs. control), and their interaction. In line with the intent‐to‐treat principle, all participants included at T1 were entered into the MMRM analyses. Model parameters were estimated using full information maximum likelihood based on all available data; therefore, participants with missing T2 data were not excluded, and the primary analyses were not restricted to completers.^6^ For the experimental group, additional MMRM analyses were conducted across T1, T2, and T3, as follow‐up data were available only in this group. All MMRM analyses included fixed effects and random intercepts. Statistical significance was set at *p* < .05 and adjusted for multiple testing using the Bonferroni correction.

To balance the two groups and to compensate for the shortcomings of a nonrandomized sample, we calculated a propensity score for each participant as the predicted probability of choosing the experimental group using available data via logistic regression analyses.^7^ As a result, each participant received a score that could vary between 0 (control) and 1 (experimental) (for a more detailed explanation of the procedure, see Müller et al., 2025). This calculated propensity score was then used as a covariate in the MMRM analyses to account for potential between‐group differences arising from the nonrandomized design.

We calculated effect sizes as Cohen's *d* for repeated measures (Cohen, 1988) for within‐group comparisons at different time points. For between‐group comparisons at T2, we calculated Cohen's *d* based on the MMRM estimates.

### Sample size

The sample size was determined a priori based on the power requirements for the primary outcome of the larger project from which this study is derived. As detailed in the associated clinical publication (Müller et al., 2025), the power analysis targeted mood, assessed with the Positive and Negative Affect Schedule Short Form (PANAS‐SF; Mackinnon et al., 1999; Watson et al., 1988). The analysis, which aimed to detect a medium effect with 80% power and a 5% alpha error, indicated a required sample size of at least *N* = 48 participants per group. This target was met for the present study.

## RESULTS

### Study participants

**FIGURE 1:**
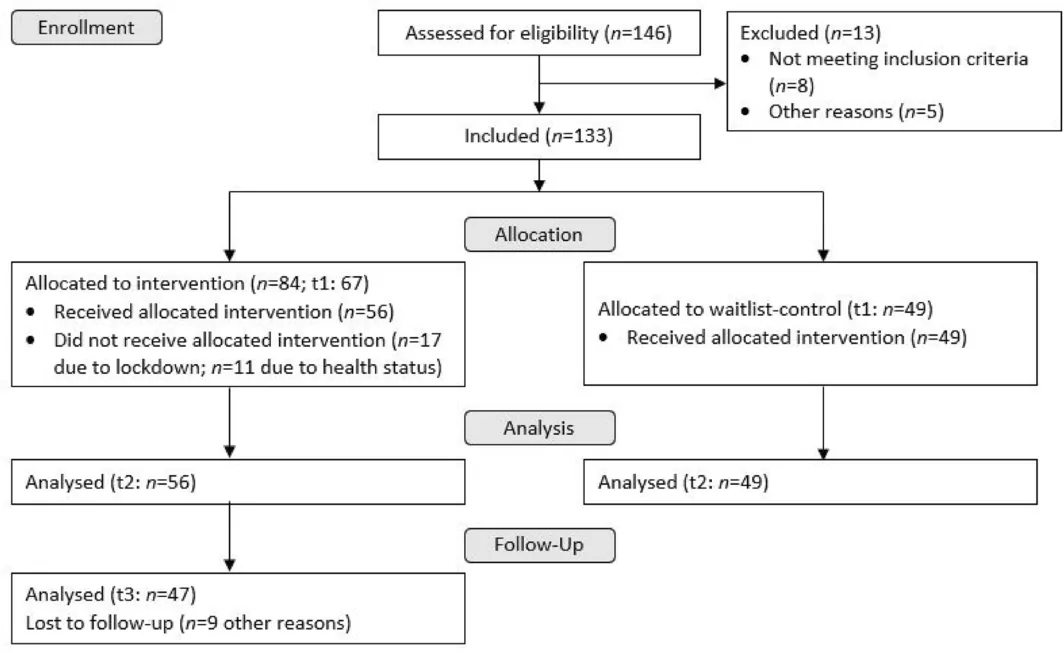
Participant flow chart.

We initially screened 146 individuals for eligibility (see Figure 1). We excluded 13 individuals (9%) for not meeting inclusion criteria or other unspecified factors and assigned the remaining 133 participants to either the experimental (*n* = 84) or control (*n* = 49) group.

Due to COVID‐19 lockdowns, 17 participants scheduled for intervention in October 2020 could not continue, reducing the experimental group to 56. For the follow‐up 3 months after the intervention, we contacted participants in the experimental group. We achieved an 84% completion rate, reflecting high participant retention. In the control group, all 49 participants completed both T1 and T2 assessments and subsequently participated in the shortened version of the intervention.

Table 1 presents sociodemographic and psychometric data at T1. To formally assess a key potential confound, a targeted post hoc analysis was conducted to check for baseline differences in previous experience with nature or mindfulness training. The analysis revealed no significant difference between the experimental and control groups (*χ* ^2^[1, *N* = 116] = 0.71, *p* = .400). Furthermore, as part of our pre‐registered sensitivity analyses for the project's primary outcome, “previous experience” did not emerge as a significant moderator of the intervention's effects on mood (see Müller et al., 2025). Thus, this variable did not appear to confound our analyses.

**TABLE 1:** Sociodemographic and psychometric data of the sample at T1 (*N* = 116) with percentages or means (95% CIs).

	Experimental group (*n* = 67)	Control group (*n* = 49)
Gender	86.6% female, 13.4% male	71.4% female, 28.6% male
Age (in years)	48.7 (37.5–59.9, min = 24, max = 68)	42.8 (25.6–60.0, min = 18, max = 85)
Parental status	74.6% yes, 25.4% no	51.0% yes, 49.0% no
Previous experience with nature or mindfulness training	40.3% yes, 59.7% no	32.7% yes, 67.3% no
Nature‐based mindfulness	5.01 (4.80–5.23)	5.17 (4.98–5.36)
Emotional affinity toward nature	5.15 (4.94–5.36)	4.88 (4.60–5.16)
Awareness of environmental threats	5.43 (5.27–5.59)	5.59 (5.41–5.77)
Internal attribution of responsibility for nature conservation	5.01 (4.79–5.24)	5.23 (4.96–5.49)
External attribution of responsibility for nature conservation	5.35 (5.15–5.55)	5.56 (5.32–5.80)
Pro‐environmental intentions	4.66 (4.46–4.87)	4.86 (4.62–5.10)
Pro‐environmental behavior	4.90 (4.69–5.11)	4.65 (4.40–4.89)
Recent time spent in nature	4.72 (4.47–4.98)	4.85 (4.52–5.19)

*Note*: Variables were measured on a 6‐point Likert scale (1 = *does not apply at all*, 6 = *fully applies*).

### Outcomes

MMRM included time, group, and their interactions as fixed factors. In addition, we included a random intercept in the analyses and a propensity score as a covariate to account for between‐group differences due to nonrandomized grouping. Consistent with hypotheses (H_1a_ and H_1b_), significant interactions between time (T1–T2) and group were expected for attitude and behavioral measures. Hypotheses H_2a_ and H_2b_ predicted significant effects of time (T1–T3) within the experimental group.

#### Changes in nature connection measures

Regarding changes in nature connection measures from pre‐intervention (T1) to post‐intervention (T2), the MMRM revealed significant Time × Group interactions for nature‐based mindfulness (*F*[1, 104.38] = 7.11, *p* = .009) and emotional affinity toward nature (*F*[1, 104.87] = 4.47, *p* = .037). Follow‐up analyses exploring these interactions showed that the experimental group exhibited significant improvements from T1 to T2 in nature‐based mindfulness (*d* = 0.36; see Figure S1A) and emotional affinity toward nature (*d* = 0.55; see Figure S1B). In contrast, the control group showed no significant changes during this period for nature‐based mindfulness (*p* = .277, *d* = 0.16) or emotional affinity toward nature (*p* = .348, *d* = 0.13). Comparing the groups at T2, we found a medium effect (*d*_between_ = 0.44). Full details, including mean changes and confidence intervals, are presented in Table 2.

**TABLE 2:** Changes in outcomes between T1 and T2 for the two groups (experimental vs. control), with effect sizes.

	Experimental group (*n* = 56)				Control group (*n* = 49)			
	T1	T2	*d*_within_	*p*	T1	T2	*d*_within_	*p*
	M (95% CI)	M (95% CI)			M (95% CI)	M (95% CI)		
Nature‐based mindfulness	4.91 (4.79–5.03)	5.12 (5.00–5.24)	0.36	.007	4.99 (4.85–5.13)	4.90 (4.76–5.04)	0.16	.277
Emotional affinity toward nature	4.83 (4.70–4.96)	5.20 (5.07–5.33)	0.55	<.001	4.73 (4.58–4.88)	4.82 (4.67–4.97)	0.13	.348
Awareness of environmental threats	5.63 (5.55–5.71)	5.43 (5.34–5.52)	0.39	.003	5.51 (5.41–5.61)	5.62 (5.52–5.72)	0.22	.129
Internal attribution of responsibility for nature conservation	4.82 (4.69–4.95)	5.01 (4.88–5.14)	0.27	.044	5.19 (5.04–5.34)	5.06 (4.91–5.21)	0.17	.239
External attribution of responsibility for nature conservation	5.23 (5.11–5.35)	5.38 (5.26–5.50)	0.24	.075	5.43 (5.29–5.57)	5.32 (5.18–5.46)	0.17	.228
Pro‐environmental intentions	4.52 (4.41–4.63)	4.59 (4.47–4.71)	0.15	.271	4.84 (4.71–4.97)	4.76 (4.63–4.89)	0.15	.310
Pro‐environmental behavior	4.66 (4.54–4.78)	4.89 (4.77–5.01)	0.35	.009	4.57 (4.43–4.71)	4.78 (4.64–4.92)	0.32	.028
Recent time spent in nature	4.59 (4.43–4.75)	4.89 (4.72–5.06)	0.28	.042	4.69 (4.50–4.88)	4.68 (4.49–4.87)	0.01	.931

*Note*: T1 = immediately preceding the initial session, T2 = immediately following the final session. The values are estimated marginal means (EMMs) from linear mixed models with repeated measures, adjusted for the propensity score as a covariate. Consequently, these model‐based estimates differ from the unadjusted, observed means presented in Tables 1 and 3.

Longitudinal analysis within the experimental group, including the 3‐month follow‐up (T3), demonstrated significant effects of time for both nature‐based mindfulness (*F*[2, 77.17] = 4.40, *p* = .016, *d* = 0.38) and emotional affinity toward nature (*F*[2, 78.27] = 8.17, *p* = .001, *d* = 0.43). For both measures, improvements observed between T1 and T2 remained stable at T3 compared to T1, indicating a sustained intervention effect (see Table 3 and Figure S1A,B).

**TABLE 3:** Changes in outcomes between T1, T2, and T3 for the experimental group, with effect sizes.

	T1	T2	T3	*d* ^(T1–T2)^	*p*	*d* ^(T2–T3)^	*p*	*d* ^(T1–T3)^	*p*
	M (95% CI)	M (95% CI)	M (95% CI)						
Nature‐based mindfulness	4.78 (4.66–4.90)	4.99 (4.87–5.11)	5.10 (4.97–5.23)	0.34	.013	0.18	.222	0.38	.007
Emotional affinity toward nature	4.70 (4.58–4.82)	5.07 (4.94–5.20)	5.07 (4.94–5.20)	0.51	<.001	0.01	.972	0.43	.003
Awareness of environmental threats	5.57 (5.49–5.65)	5.37 (5.28–5.46)	5.41 (5.32–5.50)	0.43	.006	0.09	.588	0.32	.029
Internal attribution of responsibility for nature conservation	4.78 (4.66–4.90)	4.97 (4.84–5.10)	4.94 (4.80–5.08)	0.24	.076	0.04	.777	0.17	.218
External attribution of responsibility for nature conservation	5.11 (5.00–5.22)	5.27 (5.15–5.39)	5.08 (4.95–5.21)	0.21	.110	0.26	.074	0.03	.798
Pro‐environmental intentions	4.51 (4.41–4.61)	4.59 (4.48–4.70)	4.61 (4.50–4.72)	0.01	.337	0.03	.814	0.16	.262
Pro‐environmental behavior	4.62 (4.51–4.73)	4.85 (4.74–4.96)	4.99 (4.87–5.11)	0.36	.007	0.22	.138	0.53	<.001
Recent time spent in nature	4.59 (4.43–4.75)	4.89 (4.72–5.06)	4.90 (4.73–5.07)	0.29	.033	0.14	.332	0.34	.016

*Note*: T1 = immediately preceding the initial session, T2 = immediately following the final session, T3 = 3 months after the last session. The values are estimated marginal means (EMMs) from linear mixed models with repeated measures, adjusted for the propensity score as a covariate. Consequently, these model‐based estimates differ from the unadjusted, observed means presented in Tables 1 and 2.

#### Changes in environmental cognition measures

Regarding *attributions of responsibility* for nature conservation, MMRM analysis revealed significant interactions between time (T1–T2) and group for both internal (*F*[1, 106.19] = 5.07, *p* = .026) and external attributions (*F*[1, 104.16] = 4.45, *p* = .037). Follow‐up analyses exploring these interactions showed that the experimental group exhibited a significant increase in internal attributions from T1 to T2 (*p* = .044, *d* = 0.27; see Figure S1C) and a tendency toward an increase in external attributions (*p* = .075, *d* = 0.24; see Figure S1D). In contrast, the control group showed no significant changes in these attributions over the same period (*p* = .239, *d* = 0.17 and *p* = .228, *d* = 0.17, respectively). Comparing the groups at T2, we found small effect sizes (*d*_between(internal)_ = 0.10; *d*_between(external)_ = 0.12).

Longitudinal analysis within the experimental group (T1–T3) found no significant effects of time for either internal (*F*[2, 82.74] = 1.67, *p* = .194, *d* = 0.17) or external (*F*[2, 103.82] = 2.52, *p* = .085, *d* = 0.03) attributions of responsibility.

For *awareness of environmental threats*, MMRM analysis from T1 to T2 also revealed a significant Time × Group interaction (*F*[1, 105.93] = 9.93, *p* = .002). Follow‐up analyses exploring this interaction indicated that, contrary to expectations (H_1a_), participants in the experimental group experienced a significant decrease in awareness of environmental threats from T1 to T2 (*p* = .003, *d* = 0.39; see Figure S1E). The control group, in contrast, showed no significant change in this measure during this period (*p* = .129, *d* = 0.22) with a medium effect size between the groups (*d*_between_ = 0.48).

Longitudinal analysis (T1–T3) within the experimental group showed that this decrease in awareness persisted, with levels remaining significantly lower at T3 compared to T1 (*F*[2, 93.98] = 4.63, *p* = .012, *d* = 0.32).

#### Changes in behavioral measures

For *pro‐environmental intentions*, we found no significant results in either group from T1 to T2 (*F*[1, 104.55] = 2.25, *p* = .137). Correspondingly, follow‐up analyses of within‐group changes showed no significant change in pro‐environmental intentions from T1 to T2 for either the experimental group (*p* = .271, *d* = 0.15) or the control group (*p* = .310, *d* = 0.15; see Figure S1F). Comparing the groups at T2, we found a small effect (*d*_between_ = 0.35).

Longitudinal analysis within the experimental group (T1–T3) also indicated no significant effects of time for pro‐environmental intentions (*F*[2, 84.37] = 0.77, *p* = .468, *d* = 0.16).

Regarding *PEB*, there was no significant Time × Group interaction from T1 to T2 (*F*[1, 104.97] = 0.03, *p* = .867), suggesting that the NBMI did not have a unique immediate effect on PEB compared to the control group. However, analyses of within‐group changes revealed significant positive changes in PEB from T1 to T2 for both the experimental group (*p* = .009, *d* = 0.35) and the control group (*p* = .028, *d* = 0.32; see Figure S1G). Again, we found a small effect comparing the two groups (*d*_between_ = 0.21).

Longitudinal analysis of PEB within the experimental group (T1–T3) indicated a significant overall effect of time (*F*[2, 77.53] = 7.62, *p* = .001). Specifically, participants demonstrated significantly more PEB at T3 compared to T1, with a medium effect size (*d* = 0.53), supporting H_2b_ regarding PEB changes.

For *recent time spent in nature* from T1 to T2, MMRM analysis indicated no significant Time × Group interaction (*F*[1, 100.82] = 2.17, *p* = .114). Despite the nonsignificant interaction, analyses of within‐group changes showed that the experimental group reported spending significantly more time in nature at T2 compared to T1 (*p* = .042, *d* = 0.28), whereas the control group reported no significant change over this period (*p* = .931, *d* = 0.01; see Figure S1H). Comparing the groups at T2, we found a small effect (*d*_between_ = 0.27).

Additionally, in the longitudinal analysis (T1–T3), individuals who participated in the NBMI spent significantly more time in nature at T3 than they did prior to it (T1) (*F*[2, 97.78] = 3.35, *p* = .039, *d* = 0.34).

## DISCUSSION

This study investigated the impact of an NBMI on pro‐environmental attitudes and PEBs within a general population sample over a 15‐week period. By integrating mindfulness practices with guided environmental immersion, the intervention addressed key psychological mechanisms—particularly emotional affinity toward nature—to significantly enhance pro‐environmental attitudes and foster increased PEB over time. The combination of present‐moment awareness and nature contact reinforces ecological stewardship even when environmental outcomes are not the primary focus of the intervention. Building upon prior research, these findings address critical limitations in the literature regarding specific populations and short‐term outcomes, providing nuanced insights into the pathways through which NBMIs foster sustainable attitudes and behaviors.

### Discussion of the main findings

Our study aimed to test two primary hypotheses regarding the effects of the NBMI. We predicted that the experimental group would show significantly greater improvements in environmental attitudes (nature connection and environmental cognitions; H_1a_) and pro‐environmental intentions and PEB (H_1b_) compared to the control group between T1 and T2. H_2a_ and H_2b_ examined the persistence of these enhancements at the 12‐week follow‐up (T3).

#### Impact on nature connection measures

Consistent with H_1a_, participants in the experimental group significantly improved in nature‐based mindfulness and emotional affinity toward nature from T1 to T2, compared to the control group. These findings align with literature showing that mindfulness and nature exposure deepen affective and cognitive connections to the natural world (Howell et al., 2011; Nisbet et al., 2019; Schutte & Malouff, 2018). The observed small to medium effect sizes highlight the intervention's capacity for meaningful psychological shifts, fostering present‐moment awareness of natural stimuli (Kabat‐Zinn, 1994) and cultivating deeper affective bonds, consistent with proposed mechanisms of mindfulness in nature (sensory awareness, decentering, and openness; Bishop et al., 2004; Brown & Ryan, 2003).

Theoretically, these results reinforce the critical role of affective and perceptual connection in driving pro‐environmental action, supporting models positing that deep emotional bonds and felt connection to nature are powerful motivators for conservation (Frantz & Mayer, 2014; Ives et al., 2018). Practically, this suggests that NBMIs are a promising approach for cultivating a deep, sustained connection with nature as a foundational element for environmental stewardship.

A key finding of this study is the durability of these psychological shifts; longitudinal analysis confirmed that these improvements in nature‐based mindfulness and emotional affinity were sustained at T3 compared to T1, indicating a lasting effect of the intervention on participants' sense of connection with nature, which supports H_2a_.

#### Impact on environmental cognition measures

The intervention also impacted measures of environmental cognition, as predicted by H_1a_, showing significant increases in both internal and external attributions of responsibility for nature conservation at T2 in the experimental group. This pattern in both types of responsibility attributions is particularly notable, suggesting that the intervention may foster a more comprehensive understanding of environmental challenges. This includes recognizing the importance of both personal agency and the collective action necessary for addressing global issues like climate change, as highlighted in the UN SDG 13 (Climate Action). However, longitudinal analysis revealed no sustained increase in responsibility attributions at T3, thus not supporting H_2a_ for these measures.

An unexpected finding, also related to H_1a_'s focus on environmental attitudes, was a significant decrease in awareness of environmental threats in the experimental group from T1 to T2, persisting at T3 (not supporting H_2a_ here). Although seemingly counterintuitive for pro‐environmentalism, this may reflect a complex interplay between mindfulness and environmental cognition. One plausible interpretation is that the NBMI's focus on nature's inherent value shifted attention from abstract, overwhelming global threats to immediate, care‐based appreciation. This form of appreciation is defined not by cognitive beliefs about environmental problems but by an affective, experiential sense of oneness and belonging to the natural world as a community (Mayer & Frantz, 2004). This reflects a transition from threat‐based to care‐based motivation. Alternatively, increased mindfulness might have reduced climate‐related anxiety or cognitive dissonance, fostering a more balanced stance (Brown & Ryan, 2003). The intervention could also have implicitly encouraged a focus on local, tangible nature experiences over abstract global threats, leading to reduced self‐reported “awareness” of the latter.

This warrants further investigation, particularly as recent research complicates the relationship between nature connection, threat awareness, and well‐being. For instance, a meta‐analysis by Gago et al. (2024) confirmed that climate anxiety is moderately associated with lower psychological well‐being, suggesting that high levels of threat awareness can be detrimental. Furthermore, Wullenkord et al. (2024) found that high nature connectedness can act as a vulnerability factor, making individuals more susceptible to impairing forms of climate anxiety, possibly because they are more sensitive to nature's degradation. Seen in this light, an intervention that fosters a positive, care‐based connection to nature while reducing overwhelming threat awareness might represent a psychologically adaptive pathway to pro‐environmental action.

#### Impact on behavioral measures

Regarding PEB, the findings were mixed in the short term but showed a delayed effect, partially supporting H_1b_ and H_2b_. Although both groups showed significant positive changes in PEBs from T1 to T2, no significant interaction effect meant the NBMI did not exclusively account for immediate changes, so H_1b_ was not fully supported for PEB in the short term. General PEB increases might stem from external factors like seasonal changes, study sensitization, or broader societal trends.

However, the experimental group's longitudinal analysis revealed a significant increase in PEBs at T3 compared to T1 (medium effect size), supporting H_2b_'s delayed but sustained impact. Participants also reported significantly more time in nature at T3. This pattern, where connection and nature contact precede later PEB changes, aligns with a plausible indirect pathway: The NBMI fostered deeper nature connection, encouraging greater engagement with natural environments over time, which in turn supported PEBs (Martin et al., 2020). Previous studies support this mechanism, indicating that improved nature connectedness and increased nature contact are key mechanisms linking mindfulness interventions to behavioral outcomes, often emerging over time rather than immediately post‐intervention (Capizzi & Kempton, 2023; Hanley et al., 2020; Richter & Hunecke, 2022). This delayed effect could explain the lack of distinct T1–T2 PEB differences from the control group; translating increased connection and attitudes into consistent behavior may take time (Geiger et al., 2020; Le Houcq Corbi et al., 2024).

Theoretically, these longitudinal results, particularly the delayed behavioral effects potentially mediated by nature connection and increased time in nature, add valuable nuance to the literature on mindfulness and environmentalism by helping to explain mixed findings from studies with different and often shorter evaluation periods. For instance, studies assessing effects immediately after 4‐week interventions have reported both positive (Ray et al., 2021) and even negative (Le Houcq Corbi et al., 2024) impacts on PEB, whereas other studies found no direct behavioral effects immediately following an 8‐week program (Geiger et al., 2020). Crucially, two of these studies lacked long‐term follow‐up. By tracking participants for 12 weeks post‐intervention, our finding of a delayed effect adds a critical temporal dimension that may help reconcile these seemingly contradictory results.

This proposed pathway, in which enhanced nature connection serves as a foundational mechanism driving subsequent PEB, builds on the robust, meta‐analytic evidence establishing a strong cross‐sectional link between nature connectedness and PEB (Barragan‐Jason et al., 2022; Mackay & Schmitt, 2019; Whitburn et al., 2020). Although this body of evidence is largely based on correlational data, our findings provide initial support for the temporal sequence of this relationship. This suggests that an enhanced connection to nature may indeed serve as a foundation for later pro‐environmental action. Mechanistically, our results empirically validate the proposition that mindfulness emerges from adaptive engagement with immediate‐return environments, as environmental models of mindfulness suggest (Barbaro & Pickett, 2016; van Gordon et al., 2018).

Practically, these findings suggest that environmental education and conservation initiatives could effectively incorporate mindfulness techniques within natural settings to build this foundational connection, recognizing that behavioral change may manifest over time. Programs should prioritize fostering nature connection, viewing increased time in nature as both an outcome and a facilitator of further PEB. Self‐reported PEB measures, however, may not perfectly converge with objective actions.

The null effect on pro‐environmental intentions (not supporting H_1b_ or H_2b_ for this measure) contrasts with some mindfulness‐behavioral activation studies. This dissociation suggests that NBMI‐induced PEBs may operate through implicit, habit‐based pathways rather than deliberate intentionality (Thiermann et al., 2020), supported by participants' increased habitual time in nature. This mirrors findings by Geng et al. (2015) and Maran et al. (2021), suggesting that mindfulness effectively modifies automatic environmental behaviors over prior intentions.

These findings gain broader significance when viewed through the lens of the SDGs. Specifically, the sustained increase in PEB and responsibility attributions directly supports the objectives of SDG 13 (Climate Action). Simultaneously, by deepening nature connection—a construct fundamental to psychological flourishing—the intervention aligns with SDG 3 (Good Health and Well‐being). Consequently, NBMIs emerge as valuable “co‐benefit” solutions, harmonizing the pursuit of individual well‐being with the urgent necessity of planetary preservation.

### Strengths, limitations, and future research directions

This study offers several methodological and conceptual strengths. Its longitudinal design with a 3‐month follow‐up directly responds to recent calls from major reviews for research that moves beyond short‐term outcomes to assess the long‐term efficacy of interventions (Sheffield et al., 2022) and to better understand the psychological processes through which nature connection is formed and sustained (Lengieza & Swim, 2021). By doing so, it addresses a critical gap in the NBMI literature, which has predominantly focused on immediate effects (Capizzi & Kempton, 2023; Djernis et al., 2023). Including a general population sample enhances ecological validity. The multidisciplinary intervention delivery team (psychologists, psychotherapists, and nature educators) ensured fidelity to both mindfulness principles and ecological pedagogy. The high retention rate (84% follow‐up) supports the reliability of the longitudinal findings.

Furthermore, although the study employed a nonrandomized design due to its implementation within a real‐world community program, we took several rigorous steps to mitigate potential bias. First, all main analyses used propensity scores to statistically control for observed baseline differences between the groups. Second, a targeted post hoc analysis confirmed no significant baseline difference on the key potential confound of “previous experience.” Finally, our pre‐registered sensitivity analyses for the project's primary outcome (mood) showed that this variable did not moderate the intervention's effect. Together, these measures strengthen the causal inferences drawn from the results.

However, several limitations warrant consideration, pointing to crucial future research directions. The primary limitation remains the nonrandomized design. Although we statistically controlled for observed confounders, the risk of selection bias from unmeasured variables (e.g. pre‐existing environmental values and motivation to participate) cannot be entirely ruled out. Future research should employ randomized controlled trial designs to strengthen causal inferences and enhance internal validity. Although removing the upper age limit improved demographic diversity, the recruitment from a biosphere region via channels like adult education centers likely attracted a self‐selected cohort with a pre‐existing interest in nature or mindfulness, potentially limiting the generalizability of the findings to the broader public. Therefore, future research should aim to replicate these findings using random sampling in populations with no declared pre‐existing engagement in environmental or mindfulness programs.

Methodologically, relying on self‐reported measures risks social desirability bias, particularly for PEB assessments. Relatedly, a further limitation is the use of a brief, self‐developed PEB scale. Although internally consistent, the scale was intentionally designed to be general—assessing an overall behavioral tendency rather than specific actions. We acknowledge that this approach lacks the dimensional coverage of validated, multifaceted instruments. Therefore, we recommend that future studies complement such general measures with more specific or objective metrics (e.g. ecological footprint tracking and observed behaviors) for a more comprehensive assessment. The NBMI's structure (three 4‐h sessions) and follow‐up duration, while demonstrating sustained effects, may not capture longer term trajectories or dose–response relationships. Longer follow‐up periods, potentially extending to multi‐year assessments or utilizing ecological momentary assessment (Lohani & Blodgett, 2025), are crucial for evaluating the maintenance and long‐term trajectories of both nature connection and PEBs. Pandemic‐related restrictions during implementation (e.g. reduced group sizes and specific activity modifications) may also have attenuated effect magnitudes or influenced participant experience.

The unexpected decrease in environmental threat awareness post‐intervention merits further exploration, particularly to establish whether it reflects adaptive decentering from climate anxiety or problematic disengagement from systemic issues. Future research should utilize qualitative methods or alternative assessment tools to capture these nuanced changes in environmental cognition and emotional responses to examine their relationship with changes in nature connection and anxiety.

Although the observed pattern of our longitudinal findings, in which sustained attitude changes and increased nature contact precede later increases in PEB, is consistent with an indirect pathway, a formal mediation analysis was not conducted. This is because such an analysis was not part of our pre‐registered plan and performing it post hoc would shift the research from confirmatory to exploratory. Instead, our study provides crucial longitudinal evidence that establishes the temporal precedence necessary for such a causal chain. Therefore, we strongly recommend that future research build on these findings by formally testing this proposed indirect pathway using advanced longitudinal modeling techniques on larger samples to confirm the mediating roles of enhanced nature connection and increased time spent in nature.

Geographic specificity (UNESCO Biosphere Region Berchtesgadener Land, Germany) raises questions about cultural and ecological generalizability, though the biosphere's representative habitats enhance translational potential. Conducting studies in diverse cultural, environmental, and demographic contexts is critical to enhance generalizability and understand the potential cultural specificity of the relationship between nature connection, mindfulness, and environmental engagement.

Finally, a key methodological challenge involves disentangling the precise mechanisms of change. On a construct level, the potential conceptual overlap between mindfulness and nature connectedness constructs risks inflated effect sizes, and future work should employ discriminant validity analyses to address this. Similarly, on an intervention level, the multicomponent nature of the NBMI makes it difficult to determine the core effective mechanism. By design, our intervention integrated mindfulness practices with nature contact, and this pragmatic approach does not allow us to disentangle the independent effects of each component. Therefore, future research could employ dismantling study designs to isolate the relative contribution of mindfulness versus nature contact to the observed outcomes.

Beyond these specific limitations, future studies should also aim for larger sample sizes to enhance statistical power and improve generalizability. Exploring different NBMI formats, durations, and delivery modes is essential, including integrating digital platforms for broader accessibility and scalability. Research should also examine potential moderators of intervention effectiveness, such as baseline nature connectedness, prior nature experiences, personality traits, or baseline environmental attitudes, to identify which individuals or groups might benefit most from NBMIs.

### Conclusion

In conclusion, the key contribution of this study is its longitudinal evidence demonstrating that an NBMI not only enhances pro‐environmental attitudes but, more importantly, fosters durable changes that translate into increased PEB over time. These findings highlight the potential of NBMIs to cultivate the psychological foundation for environmental stewardship, offering a promising approach to sustainability challenges by strengthening emotional connections with nature. As discussed, this contributes to both individual well‐being (aligned with UN SDG 3: Good Health and Well‐being) and planetary health (aligned with UN SDG 13: Climate Action; Lee et al., 2016). The sustained effects observed in this study indicate that even relatively short (3‐week) NBMIs can have lasting positive impacts on individuals' relationships with nature and their engagement in PEBs.

## ETHICS STATEMENT

The study was approved by the Ethics Committee of the Catholic University of Eichstatt‐Ingolstadt (No. 029‐2020 on August 26, 2020). It has also been registered in the German Clinical Trials Register (Trial Number DRKS00023369). The Universal Trial Number (UTN) registered with the World Health Organization (WHO) is U1111‐1260‐7305.

## CONFLICT OF INTEREST STATEMENT

The authors declare no conflicts of interest.

## Supporting information


**Table S1:** All scales and items used.
**Table S2:** Comparison of T1 Characteristics between Completers and Non‐Completers (Experimental Group).
**Figure S1:** Longitudinal Trajectories of Nature Connection, Environmental Attitudes, and Behaviors.

## Data Availability

The data that support the findings of this study are available from the corresponding author upon reasonable request.

## ENDNOTES

^1^
The study was approved by the Ethics Committee of Catholic University Eichstätt‐Ingolstadt (# 029‐2020). It has also been registered in the German Clinical Trials Register (Trial Registration Number DRKS00023369) and with the World Health Organization (Universal Trial Registration Number U1111‐1260‐7305).

^2^
To mitigate potential biases associated with this design, propensity score analysis was employed to balance the groups and adjust for confounding factors in the statistical analyses (see the Outcome measures section).

^3^
Further details on the specific modules and exercises developed for this NBMI are available through the GreenCARE project resources (Verwaltungsstelle der Biosphärenregion Berchtesgadener Land/Bayerische Verwaltungsstelle Biosphärenreservat Rhön, 2023; see full reference for URL).

^4^
The requirement of an adequate tetanus vaccination was included as a standard safety precaution. As the intervention involved activities in natural environments, there was a foreseeable risk of minor injuries (e.g. cuts and scrapes) that could be contaminated with soil. This criterion aligns with universal public health recommendations in Germany from the Standing Committee on Vaccination (STIKO) and was deemed a necessary measure to ensure participant safety.

^5^
A complete list of all scales, subscales, and individual items used in this study is available in Table S1.

^6^
To assess potential attrition bias within the experimental group, we compared the baseline characteristics of participants who completed the post‐intervention assessment with those who dropped out after T1. Detailed results of this comparison are provided in Table S2.

^7^
Propensity scores were calculated using sociodemographics (gender, age, previous experience with nature/mindfulness training, and parental status) and psychometric assessments where groups differed at T1 (nature‐based mindfulness, external attribution of responsibility, positive affect, negative affect, mindfulness, self‐compassion, and social support). Whereas all these variables were included for comprehensive balancing, several psychometric measures (Positive Affect, Negative Affect, general Mindfulness, Self‐compassion, and Social Support) are not otherwise detailed in this manuscript, as they pertain primarily to the primary outcomes of the project, rather than the present study's environmental focus.
