# Supplementary material for: From connection to conservation: Nature‐based mindfulness as a pathway to pro‐environmental behaviors
Source: Appl Psychol Health Well Being. 2026 Sep 9;18(5):e70220. doi: 10.1111/aphw.70220 (PMC13559042; doi:10.1111/aphw.70220)
Supplement: Supplementary file 1 [file APHW-18-0-s001.docx]

**Table S1: All scales and items used**

**Naturbezogene Achtsamkeit (Nature-based mindfulness)**

In der Natur achte ich auf Empfindungen, wie Sonnenschein in meinem Gesicht oder Wind in meinem Haar.

Ich nehme die Natur oft sehr bewusst wahr, wie z.B. die jahreszeitliche Veränderung der Bäume oder Muster aus Licht und Schatten.

Ich nehme Gerüche und Düfte in der Natur bewusst wahr, wie z.B. eine frisch gemähte Wiese.

In der Natur fällt es mir leicht, mit meinen Gedanken und Gefühlen im Hier und Jetzt zu bleiben.

Ich nehme Geräusche in der Natur bewusst wahr, wie z.B. das Rauschen von Blättern oder eines Baches.

**Emotionale Verbundenheit mit der Natur (Emotional affinity toward nature)**

In der Natur werde ich gelassener und fühle mich aufgehoben.

Wenn ich in der Natur bin, fühle ich mich frei und unbeschwert.

Wenn ich in der Natur bin, entsteht ein Gefühl von Geborgenheit.

Ich verspüre Entspannung und ein wohliges Gefühl von Vertrautheit, wenn ich in der Natur bin.

Ich fühle eine starke Verbundenheit zur Natur, wenn ich mich in ihr befinde.

Bin ich in der Natur, empfinde ich eine besondere Bindung zu ihr.

**Bewusstsein für die Gefährdung der Natur (Awareness of environmental threats)**

Wenn sich nichts Grundlegendes verändert, wird sich die Gefährdung der Natur in den nächsten Jahren verschlimmern.

Der Eingriff der Menschen in die Natur hat bereits viele negative Folgen, z.B. Gefährdung der Tier- und Pflanzenwelt, Ressourcenverknappung.

Es ist richtig, dass das Ausmaß der Naturbelastungen durch Politik und Medien so betont wird.

**Internale und externale** **Verantwortungszuschreibung für den Schutz der Natur (Internal and external attribution of responsibility for nature conservation)**

Ich fühle mich verantwortlich, selbst aktiv zum Naturschutz beizutragen.

Ich bin mitverantwortlich dafür, den Naturschutz in unserer Gesellschaft zu fördern.

Die Regierung ist verantwortlich, etwas für den Naturschutz zu tun.

Die Industrieländer tragen Verantwortung für den Naturschutz.

**Engagementbereitschaft im Naturschutz (Pro-environmental intentions)**

*Ich bin prinzipiell bereit…*

…andere darauf aufmerksam zu machen, dass naturschützende Maßnahmen wichtig sind.

…Maßnahmen zum Schutz der Natur zu ergreifen.

…mich für Parteien bzw. deren Initiativen zu engagieren, die sich für Naturschutz einsetzen.

…Produkte zu kaufen, die für die Tier- und Pflanzenwelt verträglicher sind, auch wenn diese etwas mehr kosten, z.B. biologisch abbaubares Spülmittel.

…regelmäßig für den Naturschutz zu spenden, auch Ein-Euro-Beträge.

**Naturschützendes Verhalten (Pro-environmental behavior)**

In meinem Alltag zeige ich generell naturschützendes Verhalten.

Ich verhalte mich in vielerlei Hinsicht naturschützend.

Ich lege regelmäßig naturschützendes Verhalten an den Tag.

**Kontakt mit der Natur (Contact with nature)**

Ich habe den Großteil meiner Kindheit und Jugend in einem städtischen Gebiet verbracht.

Ich verbringe aktuell viel Zeit in der Natur.

**Table S2: Comparison of T1 Characteristics between Completers and Non-Completers (Experimental Group)**

| Variable | Completers (*n* = 56) | Non-Completers (*n* = 11) | Test Statistic | *p*-value |
| --- | --- | --- | --- | --- |
| Gender (% female) | 85.7% | 90.9% | *Χ*²(1) = 0.21 | .644^a^ |
| Parental Status (% yes) | 76.8% | 63.6% | *Χ*²(1) = 0.84 | .360^a^ |
| Previous experience with nature or mindfulness training (% yes) | 39.3% | 45.5% | *Χ*²(1) =0.15 | .703^a^ |
| Age (Mean/SD) | 50.02 (10.80) | 42.36 (11.59) | *t*(13.77) = -2.02 | .063 |
| Nature-based mindfulness (Mean/SD) | 4.76 (1.06) | 4.87 (1.06) | *t*(14.27)= 0.32 | .756 |
| Emotional affinity toward nature (Mean/SD) | 4.73 (1.11) | 4.58 (1.09) | *t*(14.32) = -0.42 | .683 |
| Awareness of environmental threats (Mean/SD) | 5.59 (0.63) | 5.48 (0.62) | *t*(14.29) = -0.51 | .618 |
| Internal attribution of responsibility for nature conservation (Mean/SD) | 4.77 (1.00) | 4.82 (0.84) | *t*(15.98) = 0.18 | .863 |
| External attribution of responsibility for nature conservation (Mean/SD) | 5.15 (0.95) | 4.91 (1.09) | *t*(13.17) = -0.69 | .503 |
| Pro-environmental intentions (Mean/SD) | 4.48 (0.83) | 4.65 (0.87) | *t(*13.80) = 0.61 | .550 |
| Pro-environmental behavior (Mean/SD) | 4.61 (0.85) | 4.67 (0.99) | *t*(13.05) = 0.18 | .862 |
| Recent time spent in nature (Mean/SD) | 4.43 (1.31) | 4.64 (1.50) | *t*(13.29) = 0.43 | .672 |

*Note*. Percentages represent the proportion of participants within each group (Completers vs. Non-Completers) at baseline (T1). Categorical variables were compared using Pearson's Chi-square tests.

^a^ Fisher’s exact test was used due to small cell frequencies (expected *n* < 5).

Welch’s t-tests were used for continuous variables to account for unequal group sizes and potential variance heterogeneity. No significant baseline differences were found between completers and non-completers across any demographic or psychometric variables (all *p*s > .05).

**Figure S1: Longitudinal Trajectories of Nature Connection, Environmental Attitudes, and Behaviors**

*Note.* Changes in outcome variables across three domains: nature connection (A, B), environmental cognition (C–E), and behavior (F–H). Specifically, the panels show **(A)** nature-based mindfulness, **(B)** emotional affinity toward nature, **(C)** internal attribution of responsibility, **(D)** external attribution of responsibility, **(E)** awareness of environmental threats, and **(F)** pro-environmental intentions, **(G)** pro-environmental behavior, and **(H)** recent time spent in nature for the experimental group (solid line) and the wait-list control group (dotted line). Data points represent estimated marginal means adjusted for propensity scores. Error bars indicate 95% confidence intervals. The experimental group was assessed at baseline (T1), post-intervention (T2), and at a 3-month follow-up (T3). The control group was assessed at T1 and T2 only. Scales ranged from 1 (*does not apply at all*) to 6 (*fully applies*).
